# An fNIRS Investigation of Masculinity, Femininity, and Sex on Nonparents’ Empathic Response to Infant Cries

**DOI:** 10.3390/brainsci11050635

**Published:** 2021-05-14

**Authors:** Xinyao Ng, Li Ying Ng, Giulio Gabrieli, Atiqah Azhari, Michelle Jin Yee Neoh, Gianluca Esposito

**Affiliations:** 1 Psychology Program, Nanyang Technological University, Singapore 639818, Singapore; ngxinyao247@gmail.com (X.N.); LIYING001@e.ntu.edu.sg (L.Y.N.); giulio001@e.ntu.edu.sg (G.G.); atiqah.azhari.09@gmail.com (A.A.); michelle008@e.ntu.edu.sg (M.J.Y.N.); 2 Lee Kong Chian School of Medicine, Nanyang Technological University, Singapore 308232, Singapore; 3 Department of Psychology and Cognitive Science, University of Trento, 38068 Trento, Italy

**Keywords:** fNIRS, infant cry, masculinity

## Abstract

According to societal stereotypes, the female sex and people who are more feminine have been considered to be more empathic than males and people who are more masculine. Therefore, females and feminine individuals are expected to respond more empathically to an infant’s cries. While this hypothesis was tested using self-report scales, it has not been explored thoroughly in terms of prefrontal cortex (PFC) activity, which may be a more objective means of measuring empathy. Specifically, the medial PFC (mPFC) is involved in social cognitive processing and thus a good proxy to measure the level of empathy. This study aims to (1) assess if the empathic response, in terms of medial PFC (mPFC) activity, to infant cries differ between sexes; (2) investigate if the empathic response is moderated by levels of masculinity and femininity. Functional near-infrared spectroscopy (fNIRS) was used to measure nonparent participants’ (18 males, 20 females) mPFC response to infant cries of different pitches (high and low). The Toronto Empathy Questionnaire was used to measure trait empathy and Bem’s Sex Role Inventory was used to measure the level of masculinity and femininity. Results revealed that biological sex had no significant effect on the empathic response towards infant cries of varying pitch. Furthermore, masculinity, not femininity, was correlated with an increase in empathic response in the mPFC to high but not low-pitch infant cries. We reason that this is because of the higher aversiveness and inflicted pain associated with higher-pitched cries, which induces more emotional and physical pain that masculine individuals seek to avoid. Overall, the results suggest that greater masculinity would imply greater mentalizing and processing of empathy-related information.

## 1. Introduction

Empathy is defined as the affective sharing of experiences between two individuals. It is attributed to how our autonomic nervous systems are biologically programmed to produce similar responses when triggered by affective expressions of another member of the same species [1]. Despite not being gender-specific, it is a common belief that the female sex has a greater innate ability to be empathic, in comparison to the male sex.

Studies have consistently suggested that there is a biological basis to the female sex being more empathic than the male sex. Eisenberg and Lennon [2] demonstrated that affective role-taking, a form of empathy, was present and reliably favored women in self-reports, with longitudinal and developmental studies providing further support to these findings [3,4]. Developmental studies similarly emphasized that female, but not male, children have a propensity to cry in response to another child crying, indicating that the greater capacity for cognitive and affective empathy in females may be innate.

Neuroimaging studies reinforce this perspective by demonstrating that there are biological sex differences in the activation of neural networks responsible for processing emotions. These networks span from the frontal regions to subcortical areas [5]. Findings include (1) stronger lateralization of neural networks related to emotional processing [6], (2) differential neural activation during empathic tasks in males in comparison to females [7], (3) neural sex differences, such as the bilateral processing of emotions in both hemispheres [8], and (4) the significantly greater synaptic density of the temporal neocortex in females [9], which could enhance female capacity for socioemotional processing. Additionally, the empathizing–systemizing theory by McClure [10] and related studies showed support for the sexual dichotomy of the brain being the source of the empathic difference between biological sexes. These studies showed that females’ brains were biologically predisposed to be empathic, with females consistently outdoing males in verbal skills, empathy, security-seeking, and social skills [11]. Hence, self-report and neurological results provide a basis for the belief that females are biologically more empathic than males.

On the other hand, research into masculinity and femininity suggests that biological females are more empathic compared to biological males due to the internalization of masculine and feminine gender roles. These gender roles are biological sex-based behavioral expectations attributed to each sex [12]. From a sociological perspective, the social role theory explains that though the gender roles are flexible, it is partially constrained by individuals’ physical attributes, their related behaviors, and a myriad of factors present in society [13]. Traits and characteristics that would be expected to be possessed by a female in today’s society would be considered a feminine trait, whereas those that would be expected to be possessed by a male would be considered masculine. In line with this expectation, studies have suggested that females are more empathic because society expects feminine individuals to be more empathic [14]. Hence, females are suggested to be more empathic as they are imbued with the societal ideals of femininity, which assists them in carrying out the tasks they are expected to, such as child-raising.

Common measures of empathy used in the above-mentioned studies were self-report scales. These include examples such as The Empathy Scale, Interpersonal Reactivity Index and Questionnaire Measure of Emotional Empathy, and the Toronto Empathy Questionnaire (TEQ), all of which measure empathy through self-report scales. For this study, we chose to use the TEQ because the TEQ is a measure of empathy that was created from the underlying consensus among previous empathy scales. The TEQ also displays high convergent validity, internal consistency, and test–retest reliability with many other empathy scales [15].

Newer functional imaging studies, however, have identified brain responses associated with empathy. Empathy is known to be a common reaction towards situations such as infant cries, as the caretaker would respond empathically to reduce the distress experienced by the infant [16]. Several studies found that when female or male participants listened to infant’s cries, strong activation occurred in specific regions of the brain, for both parents and nonparents. These regions include the medial prefrontal cortex (mPFC), inferior frontal gyrus (IFG), anterior insula, and bilateral posterior cingulate, all of which are brain regions associated with empathy [17,18,19,20]. These results provide an indication that an empathic response, in terms of brain activation, was observed in both males and females when presented with infant cries.

Activation patterns of the mPFC are a suitable neurological measure of empathic response as the mPFC is a core mediator of empathy [21] by virtue of its involvement in the processing of empathy-related information [22]. Activity in the mPFC displays robust relation to empathy by its strong association with mentalization processes, the cognitive aspect of understanding the others’ mental states [23] and Theory of Mind, the ability to attribute other’s mental states (ToM) [24], which are central components of empathy. Support for the involvement of mPFC in empathy was also shown in previous studies that found mPFC activity to be positively correlated to trait empathy and prosocial behavior and acted as a mediator of the relationship between the two [25]. Hence, given the strong involvement of the mPFC in empathic responses, activity levels of the mPFC could serve as an objective measurement of empathic response.

Before an empathic response can be evoked by the empathizer, the empathic stimulus must first be perceived, such as an infant’s cries being perceived by the caretaker. Infants’ cries are known as a ‘biological siren’ [26] that serve primarily to provide infants with the means to quickly convey their distress, urgency, pain, or to alert their caretakers [27] by being perceived as aversive. Infant vocalization studies, such as the study conducted by Murray [28], found that both parents and nonparents perceived infant cries as aversive. This aversiveness of infant cries further arises from its pitch. Cries with higher pitch as compared to typical cries were perceived by parents as more sick sounding, urgent, distressing, arousing, and were associated with future infant abuse [29,30].

Despite its perceived aversiveness, infant cries elicit prosocial behaviors from caretakers to soothe the infant [31]. Although the prosocial behavior may be motivated by egotistical means to reduce the aversion induced by the cries [32], it may be attributed to empathy as well [31,33]. Previous studies found that an increase in empathy promoted greater prosocial behavior [23]. Increased mentalizing, a form of empathy, allows the empathizer to perceive the subject of empathy, the infant, as more similar to themselves. In this instance, drawing similarity to the empathizer themselves when they were crying from distress or pain increases prosocial behavior as they may be more willing to help those they view as being more similar to themselves [23,34]. Despite increasing negative emotions such as anger, an increment in the aversiveness of an infant cry may, hence, induce an increase in empathy to promote prosocial behavior. This may result in an empathic response in the brain when infant cries are heard [16].

The empathic response, however, can be conceptualized into two distinct but interrelated components, namely cognitive empathy, and emotional empathy. Cognitive empathy involves socio-cognitive perspective-taking by the empathizer [35], whereas emotional empathy entails the vicarious experience of another’s emotions [36]. Cognitive empathy was further found to be more strongly associated with prosocial behavior, whereas emotional empathy was less strongly associated [37]. Thus, the present study focuses on cognitive empathy.

Although a significant amount of research has been conducted in this domain, most of the prior investigation utilized self-report measure, thus this research aims to investigate an alternative mechanism via brain activity. At the same time, we sought to replicate previous findings and suggestions that female sex and femininity would correlate with higher levels of empathy. As such, the study hypotheses are given below.

**Hypothesis** **1** **(H1):** *Biological females respond more empathically to infant cries, based on higher mPFC activity compared to biological males, controlling for trait empathy.*

**Hypothesis** **H2a** **(H2a):** *Increased levels of femininity correlates to higher levels of empathy in response to infant cries, based on higher mPFC activity compared to lower levels of femininity.*

**Hypothesis** **H2b** **(H2b):** *Increased levels of masculinity correlates to lower levels of empathy in response to infant cries, based on lower mPFC activity compared to lower levels of masculinity.*

## 2. Materials and Methods

### 2.1. Participants

Participants were young adults who are nonparents (n = 38; 20 females (M age = 22.65 ± 1.69 years), 18 males (M age = 23.11 ± 2.35 years)).

### 2.2. Procedure

The methods and procedures of this study were approved by the ethics committee of the Nanyang Technological University. Participants completed a participant demographic survey (PDS) and the Toronto Empathy Questionnaire (TEQ) a day before the actual study to record demographic information and trait empathy. Details about the surveys are provided in the next subsection. Prior to responding to the questionnaire, written informed consent was obtained.

In the study, a series of six infant cries of high and low pitch with a duration of 15 seconds was presented. This consists of three high pitch and three low-pitch cries. Participants were presented either with high pitch followed by low pitch and high pitch again and so on, or low pitch followed by high pitch and low pitch again and so on. Within the three high-pitch cries, the order of presentation was randomized, similarly for the low-pitch cries. While the stimulus was presented, the participants’ empathic response in terms of mPFC blood oxygenation levels were measured using a non-invasive BrainSight (BrainSight, Rogue Research, Montreal, Canada) functional near-infrared spectroscopy (fNIRS) device. After the presentation of the stimuli, participants completed the Bem Sex-Role Inventory (BSRI) questionnaire.

### 2.3. Measures

#### 2.3.1. Participant Demographic Survey

Information regarding the participants’ biological sex was collected using the PDS, by participants’ indication if they were female or male at birth.

#### 2.3.2. Toronto Empathy Questionnaire

The TEQ is a self-administered 16-item empathy measure by providing a measure of empathy correlated with both cognitive and emotional empathy [15]. Respondents rated how frequently they felt in manners as described in the statements such as ‘When someone else is feeling excited, I tend to get excited too’. on 5-point Likert scales ranging from ‘never’ to ‘always’. The TEQ was highly reliable, with Cronbach’s alpha values of 0.78 in the present study, high test–retest reliability (r = 0.81), and high construct validity (r = 0.80) [38].

#### 2.3.3. Near-Infrared Spectroscopy

**Figure 1:**
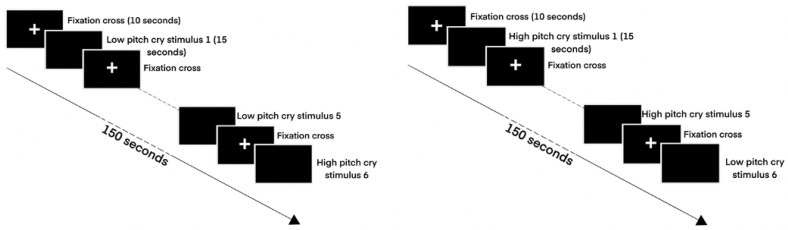
Diagram of experimental stimulus presentation orders.

**Figure 2:**
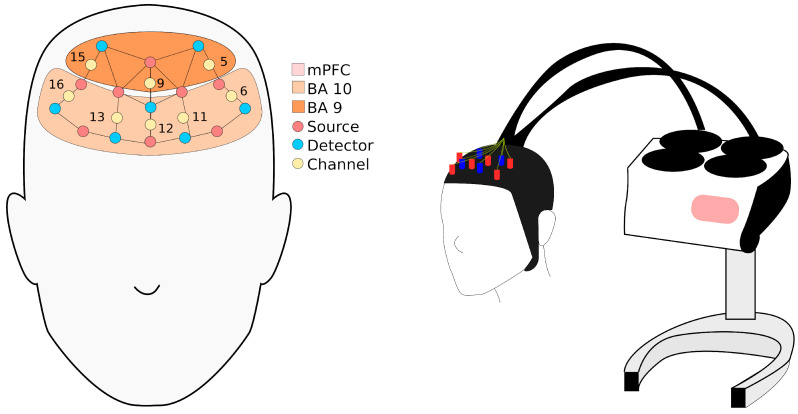
Locations of the 8 optode channels and their corresponding positions with respect to BA 9 and 10 in the mPFC and schematic view of the experimental setup.

Empathic response in terms of activity in the mPFC was measured using fNIRS. The fNIRS had a constant scan rate of 10 Hz and used LED emissions at source wavelengths of 705 nm and 830 nm. Inter-optode distance was kept within the range of 29.1 mm to 36.5 mm, as close as possible to the optimum inter-optode distance of 30 mm. BrainSight uses a 9 × 7 source-detector PFC montage, configured with a 20-channel-recording system to record PFC activation during stimulus presentation (Figure 1). Investigation of the mPFC was conducted via individual channels (i.e., channels 5, 6, 9, 11, 12, 13, 15, 16; see Figure 2) to provide a more precise perspective and understanding of specific empathy-related Brodmann areas (BA) 9 and 10 that make up the mPFC; both are crucial in emotional and cognitive empathy, with BA 10 also being involved in pain-related processing [39,40].

MATLAB software (MATLAB 9.6) and HOMER2 (v2.8) scripts were used to pre-process, analyze, and manually add stimulus onset and offset markers to the fNIRS data. A configuration file was used in the HOMER2 processing stream (hmrIntensity2OD, hmrMotionCorrectWavelet (iqr = 0.10), hmrBandpassFilt (hpf = 1.010, Ipf = 0.50), hmrOD2Conc (ppf = 6.0.6.0), hmrBlockAvg (trange = −2.0 20.0)) to automatically pre-process the data and correct motion wavelets.

#### 2.3.4. Bem Sex-Role Inventory

The BSRI [41] is a 60-item self-assessment questionnaire consisting of 3 parts, a femininity scale, masculinity scale, and a gender-neutral scale. For the purposes of this study, only the scores for masculinity and femininity scales were analyzed. Participants rated themselves on a 7-point Likert scale based on how well they think the traits represented themselves, with 1 indicating that the trait is never or almost never true and 7 indicating that the trait is almost always true. The masculinity and femininity scale consists of 20 items each, and higher scores represent higher level of masculinity or femininity, respectively. Each participant fills in the full questionnaire and obtains an individual masculinity and femininity score. The BSRI has high internal consistency, with a Cronbach’s alpha of 0.86 for the masculinity scale and 0.83 for the femininity scale based on the whole sample.

The scores were initially intended to be used for the categorization of participants into four distinct gender roles using a median split based on the results of Bem’s normative sample [41]. However, due to a highly uneven distribution of participants into different gender roles, the BSRI questionnaire scores were used as continuous measures of masculinity and femininity instead, as the BSRI measured masculinity and femininity separately as independent measures [42].

### 2.4. Infant Cry Stimuli

Three high and three low-pitch cries of a three-month-old infant taken from the Social and Affective Neuroscience Laboratory infant cry database were used as the experimental stimuli. High and low-pitch infant cries were categorized using Green et al. [43] definitions of fuss and yell, respectively. Fuss referred to low-pitch cries of shorter duration vocalizations and indicates lower distress, while yell referred to high-pitch cries of longer duration vocalizations and indicates higher distress. All infant cries were adjusted using PRAAT (Ver. 6.1.10) to remove pauses between cries, with settings adapted to analyze cry samples [44]. Pitch matching was conducted to ensure the similarity of low-pitch cries with one another and high-pitch cries with one another. Volume was adjusted to 70 dB SPL. Low-pitch cries were tuned to an average fundamental frequency of 361.92 Hz (Standard Deviation (SD) = pm 50.75 Hz) and high-pitch cries were tuned to an average fundamental frequency of 426.22 Hz (SD = pm 79.11 Hz). An independent sample t-test conducted between the low and high-pitch cries indicated that they are significantly different (*p* = 0.029). Each cry stimulus was presented once for 15 s, followed by 10 s of silence with a white fixation cross on a blank black screen (Figure 1). The cries were presented in an alternating order, high pitch followed by low pitch or low pitch followed by high pitch, with each cry being only played once per session.

### 2.5. Analytic Plan

The biological sex and BSRI scores were the between-subject factors, whereas the pitch of the infant cries was the within-subjects factor in the analysis of variance (ANOVA). The fNIRS beta values and TEQ scores were the outcome variables of interest. Preliminary and inferential ANOVA, Pearson-moment correlation, and Fisher’s exact test were conducted in R studio (Ver. 3.5.2, macOS 10.12) using R-Base (3.5.2, macOS 10.12) for the fNIRS and questionnaire data. All values obtained from the ANOVA, Pearson-moment correlation test, and Fisher’s exact test were subjected to false discovery rate (FDR) correction as well as Bonferroni’s correction when applicable.

**Figure 3:**
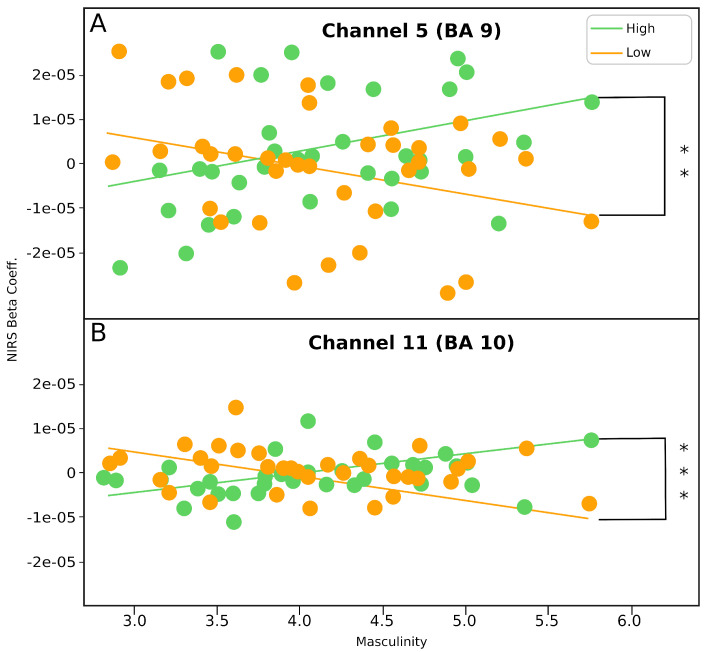
(**A**) Plot of correlation between masculinity scores and activation in channel 5 (BA 9). (**B**) Plot of correlation between masculinity scores and activation in channel 11 (BA 10). ** *p* < 0.01; *** *p* < 0.001.

Figure 3 shows a recap of the results. To examine the first hypothesis that predicts that females would respond more empathically in terms of mPFC activation towards both cry pitches, a two-way repeated measures ANOVA was conducted on the beta values computed from the fNIRS data of the mPFC with the participant’s biological sex as between-subjct factor and cry pitch as within-subject factors. A one-way ANOVA was then conducted on the TEQ scores to examine if the same hypothesis that expects females to be more empathic holds in terms of trait empathy.

To test the second hypothesis that masculinity and femininity moderate empathy, a Pearson-moment correlation was then conducted on the beta values of all mPFC channels with masculinity or femininity scores to identify the strength of the correlation between masculinity, femininity, and empathy.

## 3. Results

### 3.1. fNIRS Results

The two-way repeated measures ANOVA on biological sex and cry pitch showed no significant main effect nor interaction effects of biological sex on empathic response in terms of mPFC activity for both low and high-pitch infant cries. Since all results of the two-way repeated measures ANOVA on biological sex and cry pitch were non-significant (*p* > 0.05), FDR or Bonferroni correction was not carried out.

### 3.2. Sex Difference in Trait Empathy

The one-way ANOVA highlighted no significant differences between the TEQ scores between sexes (F(1, 36) = 3.34, *p* = 0.076, η *p*${}^{2}$ = 0.09).

### 3.3. Correlation between Femininity and mPFC Activity

No significant correlation was found when a Pearson-moment correlation test was conducted between femininity scores and mPFC activity in all channels for both low and high-pitched cries.

### 3.4. Correlation between Masculinity and mPFC Activity

Significant correlation was found in (1) channel 5, BA 9, low-pitch cry × Masculinity scores, high-pitch cry × Masculinity scores; (2) channel 11, BA 10, low-pitch cry × Masculinity scores, high-pitch cry × Masculinity scores (Table 1; Figure 3) when a Pearson-moment correlation was conducted. The results of the Fisher’s r-to-Z transformation showed that the correlations between low-pitch cry × Masculinity scores and high-pitch cry × Masculinity scores were significantly different in channels 5 (Z = −2.97, *p* = 0.00150) and 11 (Z = −3.16, *p* = < 0.001). A summary of the results of the Pearson-moment correlation test and Fisher’s r-to-Z transformation is shown in Table 1.

## 4. Discussion

The aim of the present study was to investigate the effects of biological sex, masculinity, and femininity (gender roles) on empathic neural responses, through mPFC activation, by presenting infant cries of varying pitches to nulliparous participants. Our study consisted of two hypotheses. Firstly, females were expected to display a greater empathic response, in terms of mPFC activity, in comparison to males towards both pitches of cries. Secondly, we anticipated femininity to be a moderator of empathy, with individuals of higher femininity scores displaying a greater empathic response. Lastly, we expected masculinity to be a moderator of empathy as well, with individuals of higher masculinity scores displaying a lower empathic response. The results of the study partially support the third hypothesis as masculinity moderated the empathic response towards cries of different pitches but, greater masculinity scores resulted in a higher empathic response. The first and second hypothesis, however, was not supported by our investigation as the results displayed no significant difference in empathic response between sexes neither was empathy being moderated by femininity.

The results of our study indicated that the biological sex of the listener had no significant effect on the empathic response elicited. We do not contend that biological sex has no effect on the empathic response, but rather that the participants in the present study may be similarly empathic, as reinforced by the results of the TEQ questionnaires which showed no significant sex differences in scores. This implies that between sexes in this sample, the level of trait empathy was not significantly different. Furthermore, the similar levels of activation in both sexes, observed in BA 9 and 10, reinforces that the levels of trait empathy between sexes were not significantly different as they are strongly correlated [23]. Another possible explanation for the non-significant difference in empathy between sexes may be related to the age of the participant sample. The participant sample consisted of mostly university undergraduates, nonparents, with a mean age of 22.9 years old (SD = 2.02), with a low frequency of interaction with infants. Hence, they are possibly less knowledgeable and aware of the importance of cries, the familial roles of the mother and father, and the implicit emotional connotations that are tied to the cries of an infant. The combination of being nonparents and having a low frequency of interaction with infants may have resulted in a non-significant difference in the fNIRS beta values towards infant cries [45].

Although no significant differences were found in the empathic responses of individuals of different sex, we found an association between level of masculinity and brain activity during high-pitch cry. A possible explanation could be that we found that masculinity’s association with pain threshold and tolerance [46,47] may have resulted in masculinity’s moderating effect on the empathic response elicited. The significant interaction effect between the participants’ masculinity and empathic response (Table 1) indicates that masculinity, not femininity, moderated the empathic response through BA 9 and 10, as both are strongly correlated with empathic processing [48,49], contradictory to previous studies on empathy and gender roles [13].

We contend that the result of masculinity moderating empathic response rather than femininity may be attributed to the aversive characteristic of infant cries inflicting pain on those who hear it [50]. This is indicated by the deactivation in medial BA 10 and BA 9 in the default mode network (DMN) [51], a network of brain areas, which also consists of the mPFC and other empathy-related brain regions, that are intrinsically active during resting states and are deactivated when a sensory stimulus is presented [52,53]. The DMN is deactivated in response to pain to provide cognitive resources to the lateral BA 10, which is implicated in processing and modulation of sensory pain [40]. High masculinity scores were positively correlated with a high pain threshold [54], thus, the pain of hearing the high-pitch infant cry may not be perceived to be as great in masculine individuals in contrast to individuals with low masculinity. Masculine individuals, therefore, may not require as much cognitive resource to successfully process and modulate sensory pain, allowing them to maintain activation of brain areas in the DMN to engage in empathic processing while simultaneously processing and modulating their pain without task-induced deactivation. Similarly, masculinity’s positive association with pain threshold may extend to explain why individuals with higher masculinity scores displayed significantly lower empathy towards low-pitch infant cries than high-pitch infant cries (Figure 3). A higher pain threshold in masculine individuals may suggest that the low-pitch infant cries may not exceed the pain threshold for more masculine individuals, and thus, may not be considered an aversive stimulus. Hence, the low empathic response during the low-pitch infant cry may have been due to a lack of perception of an aversive stimulus, which resulted in a reduced desire to carry out prosocial behavior as there was no negative state that needed to be reduced [16,55].

Our study demonstrates that the characteristic of infant cries, such as the pitch and the possible emotional and physical pain it inflicts, as represented by the activation in BA 10, is a potentially important factor in the understanding of empathy and care response towards infants. The novel interaction between masculinity and empathy is representative of a possible relationship between an individual’s gender role and neural substrates of empathy, especially in infant and pain-related empathic experiences. These findings shed light on the plausible effects and importance of socialization and assimilation of gender roles on the neural substrates of empathy and empathic processing. The novel discovery of masculinity’s moderation of empathic response, rather than femininity, is a potential missing link in the understanding of why males are often the perpetrators of child abuse [56,57]. The results of this study shed light on a potential sea of issues on how masculine socialization of men may be responsible for such an outcome.

Nevertheless, there are several limitations that may affect the generalizability of the findings. Firstly, only nonparent participants were examined in the present study. Parental experience may be a significant factor in empathy towards infant cries as shown by prior studies [45,58]. Secondly, due to the limitations of the fNIRS, other empathy-related subcortical regions were unable to be examined. The usage of fNIRS provides the benefit of an enhanced temporal resolution due to its high sampling rate, but it is limited in spatial resolution to the cerebral cortex [59]. Lastly, infant cries of only a limited number of different pitches were examined in this study, even though in actuality, infant cries contain a significantly larger range of pitches. Different pitches of infant cries include a multitude of different phonetic characteristics, which can be picked up to communicate a variety of information about the infant, ranging from hunger to sickness [60].

In conclusion, there is evidence of a potential relationship between infant cry pitch, empathic-related neural responses in the mPFC, and masculinity. Pain inflicted due to the aversive nature of infant cries may reduce the empathy they invoke through the task-induced deactivation of empathy and pain-related neural networks in the individual that hears the cry. This pattern of deactivation persisted regardless of the participants’ sex, which may be attributed to the youthfulness of nulliparous adults with regards to their lower frequency of interaction with infants and their unfamiliarity with motherly and fatherly familial roles. Furthermore, the level of masculinity moderated the empathic response through its association with pain threshold and tolerance, allowing sufficient cognitive resource for the simultaneous activation of empathy and pain-related neural networks. The results obtained provide a closer look at the intricate mechanisms of infant cry pitch and masculinity and perhaps the involvement of femininity in empathy. Enhanced knowledge of these mechanisms may further develop our understanding of caregiving response, infant abuse, infanticide, and a potentially crucial link between Singapore and greater Asia’s socialization of males and increasing child abuse [56].

## Figures and Tables

**Table 1:** Fisher’s r-to-Z transformation test for the effect of masculinity trait scores and high and low pitch infant cry on empathic response.

	Low-Pitch Cry × Masculinity Scores		High-Pitch Cry × Masculinity SCORES		Low-Pitch Cry Masculinity Scores × High-Pitch Cry Masculinity Scores	
	N	r	N	r	Z	*p*
Channel 5	38	−0.34	38	0.35	−2.97	0.0015 **
Channel 11	38	−0.32	38	0.41	−3.16	0.0008 ***

** *p* < 0.01, *** *p* < 0.001.

## Data Availability

Data are available online on the Data Repository of the Nanyang Technological University at the following url https://doi.org/10.21979/N9/SWQK3O (Accessed on 12 May 2021).
